# Mussel-Inspired Injectable Adhesive Hydrogels for Biomedical Applications

**DOI:** 10.3390/ijms25169100

**Published:** 2024-08-22

**Authors:** Wenguang Dou, Xiaojun Zeng, Shuzhuang Zhu, Ye Zhu, Hongliang Liu, Sidi Li

**Affiliations:** 1 School of Chemistry & Chemical Engineering, Yantai University, Yantai 264005, China; 2 Shandong Laboratory of Yantai Advanced Materials and Green Manufacturing, Yantai 265503, China

**Keywords:** mussel-inspired biomaterials, injectable hydrogels, adhesive hydrogels, biomedical applications

## Abstract

The impressive adhesive capacity of marine mussels has inspired various fascinating designs in biomedical fields. Mussel-inspired injectable adhesive hydrogels, as a type of promising mussel-inspired material, have attracted much attention due to their minimally invasive property and desirable functions provided by mussel-inspired components. In recent decades, various mussel-inspired injectable adhesive hydrogels have been designed and widely applied in numerous biomedical fields. The rational incorporation of mussel-inspired catechol groups endows the injectable hydrogels with the potential to exhibit many properties, including tissue adhesiveness and self-healing, antimicrobial, and antioxidant capabilities, broadening the applications of injectable hydrogels in biomedical fields. In this review, we first give a brief introduction to the adhesion mechanism of mussels and the characteristics of injectable hydrogels. Further, the typical design strategies of mussel-inspired injectable adhesive hydrogels are summarized. The methodologies for integrating catechol groups into polymers and the crosslinking methods of mussel-inspired hydrogels are discussed in this section. In addition, we systematically overview recent mussel-inspired injectable adhesive hydrogels for biomedical applications, with a focus on how the unique properties of these hydrogels benefit their applications in these fields. The challenges and perspectives of mussel-inspired injectable hydrogels are discussed in the last section. This review may provide new inspiration for the design of novel bioinspired injectable hydrogels and facilitate their application in various biomedical fields.

## 1. Introduction

In nature, marine mussels secrete proteinaceous glues to tightly adhere to wet rock surfaces [1]. These proteinaceous glues quickly solidify upon contact with surfaces, forming adhesive plaques featuring remarkable interfacial strength and resilience [2]. Over recent decades, extensive efforts have been made to understand the intricate compositions and adhesive mechanisms of these mussel-derived proteins [3,4,5]. These works reveal that a noncanonical amino acid, namely, 3,4-dihydroxyphenylalanine (DOPA) exists in the mussel adhesive proteins [6,7]. In the proteins on the surface of mussel adhesive plaques (Mfps 3 and Mfps 5), the contents of DOPA are higher and reach 20% and 28%, respectively [8]. This phenomenon indicates that DOPA plays an essential role in the adhesion of mussels.

**Figure 1:**
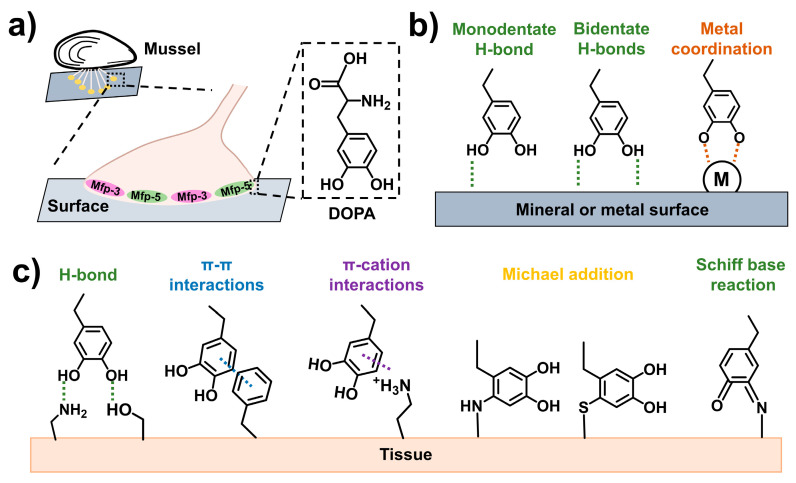
Adhesion of mussel-inspired materials. (**a**) The adhesion of marine mussels by adhesive plaques composed of DOPA. (**b**) The interactions of catechol group with mineral or metal surfaces. (**c**) The interactions of catechol group with biological tissue surfaces [9,12].

The main functional group in DOPA is the catechol group, which can form multiple covalent and noncovalent interactions with specific surfaces [9,10,11] (Figure 1). For mineral or metal surfaces, the adhesion interactions mainly include hydrogen bonding (H-bonding) and coordination interaction. For organic surfaces, especially biological tissue surfaces, more adhesion interactions may form due to the complicated components of organic surfaces. In addition to H-bonding, π-π interactions and π-cation interactions may exist. If the catechol groups are oxidized by oxidizing agents, such as sodium periodate (NaIO_4_), the catechol group will transfer to a quinone structure [12]. Under this circumstance, covalent bonding can form with the amino or sulfhydryl groups on the surface of biological tissues through Schiff base reaction or Michael addition [13]. Considering the versatile roles of the catechol group in adhesion, in recent decades, a large number of studies have introduced the catechol group into polymer chains to develop adhesives. These mussel-inspired glues exhibit desirable adhesive performance and are applied in numerous fields, including biomedical adhesives [14,15], antifouling coatings [16,17], flexible electronics [18,19], drug delivery [20,21], cell encapsulation and delivery [22,23], etc.

A hydrogel is a three-dimensional (3D) polymeric network formed by the physical or chemical crosslinking of hydrophilic polymers [5,24]. By virtue of their biological tissue-similar structure, biocompatibility, and tunable physical properties, hydrogels are widely applied in various biomedical applications [3,25,26]. Regarding the formation site, hydrogels can be divided into preformed hydrogels and in situ injectable hydrogels [27]. These two types of hydrogels have their characteristics (Table 1). In general, preformed hydrogels form in specific reactors or molds. Some complicated conditions, such as high temperature, long reaction time, and oxygen-free conditions, can be satisfied for the formation of preformed hydrogels [28]. Therefore, preformed hydrogels usually exhibit high physical performance, such as high mechanical properties, strong adhesive properties, etc. However, since preformed hydrogels are formed in molds, they find it difficult to precisely fill irregular defects [29]. In addition, for internal applications, a relatively large incision is usually required to implant the preformed hydrogel into the body. Different from preformed hydrogels, in situ injectable hydrogels utilize the sol–gel transition process to achieve a minimally invasive way of application [30]. The precursor solution of these hydrogels is injectable, and after the precursor is injected into the target site of the body, the precursor transforms into a gel state. Therefore, the injectable hydrogels can fill irregular shapes well. However, due to limited formation conditions, such as 37 °C and physiological pH, injectable hydrogels usually exhibit weak mechanical and adhesive properties compared with preformed hydrogels [31]. Nevertheless, the minimally invasive delivery method enables injectable hydrogels to be a promising tool in clinical practice [32].

**Figure 2:**
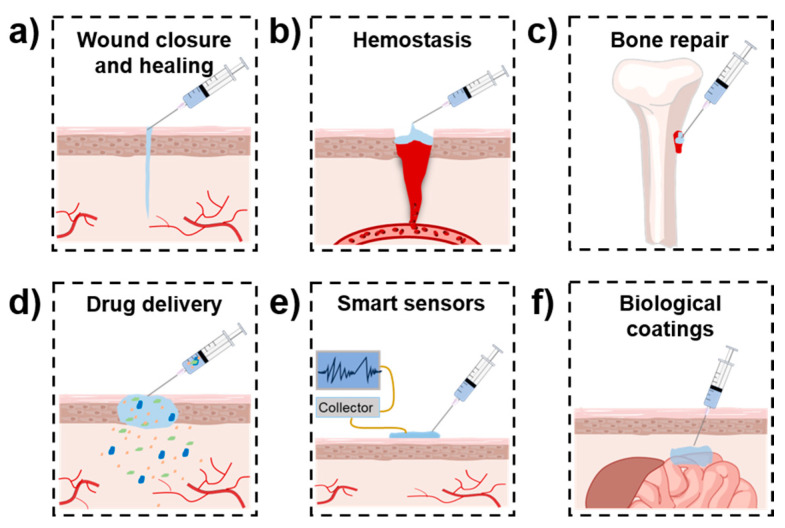
Biomedical applications of mussel-inspired injectable adhesive hydrogels. (**a**) Wound closure and healing; (**b**) Hemostasis; (**c**) Bone repair; (**d**) Drug delivery; (**e**) Smart sensors; (**f**) Biological coatings.

To improve the properties of injectable hydrogels, in recent decades, various mussel-inspired injectable hydrogels have been developed. Different from other related reviews [33,34,35], this review mainly focuses on mussel-inspired hydrogels with injectability. This type of hydrogel simultaneously possesses the minimally invasive property of injectable hydrogels and the desirable functions provided by mussel-inspired components, which may include tissue adhesiveness and self-healing, antimicrobial, and antioxidant capabilities [33]. Their fascinating properties benefit their application in numerous biomedical fields, such as wound closure and healing [36,37], hemostasis [38,39], bone repair [40,41], drug delivery [42,43], smart sensors [44], biological coatings [45], etc. (Figure 2). Typical examples are summarized in Table 2.

In the following review, we summarize the typical design strategies of these mussel-inspired injectable adhesive hydrogels. Further, we systematically overview the recently designed mussel-inspired injectable adhesive hydrogels. In addition, we mainly focus on how the specific properties of the hydrogels facilitate their application in specific fields. The challenges and perspectives of mussel-inspired injectable adhesive hydrogels are also discussed in the last section of this review.

## 2. Design Strategy of Mussel-Inspired Injectable Adhesive Hydrogels

### 2.1. Incorporation of Catechol Group into Polymers

**Figure 3:**
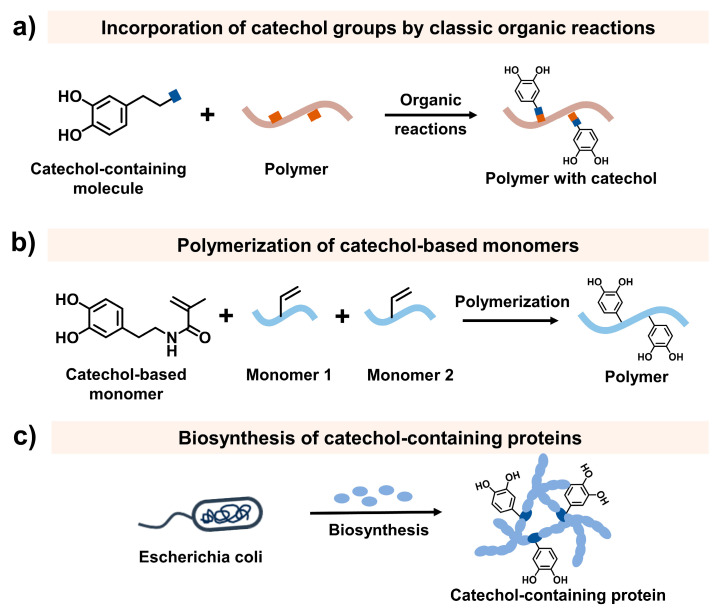
Typical strategies for incorporation of catechol groups into polymers. (**a**) Incorporation of catechol group by classic organic reactions. (**b**) Polymerization of catechol-based monomers. (**c**) Biosynthesis of catechol-containing proteins.

The yield of extraction of mussel adhesive proteins is extremely low [46]. Therefore, it is difficult to directly use natural mussel adhesive proteins to develop injectable adhesive hydrogels. Due to the essential role of DOPA in mussel adhesion, mussel-inspired injectable adhesive hydrogels are usually developed by engineering the catechol group into the gel network. Incorporation of the catechol groups into the polymers is usually the first step to developing mussel-inspired injectable hydrogels. During recent decades, many methods have been developed to incorporate the catechol group into polymers [4,47], including the incorporation of the catechol group by classic organic reactions, polymerization of catechol-based monomers, and biosynthesis of catechol-containing proteins (Figure 3).

#### 2.1.1. Incorporation of Catechol Groups by Classic Organic Reactions

**Figure 4:**
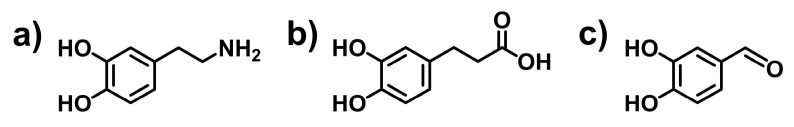
The chemical structures of typical commercial DOPA derivatives. (**a**) 3,4-dihydroxyphenethylamine; (**b**) 3-(3,4-dihydroxyphenyl) propionic acid; (**c**) 3,4-dihydroxybenzaldehyde.

Since there are many commercial DOPA derivatives (Figure 4), the incorporation of the catechol group into polymers by classic organic reactions is easy to perform. The coupled reaction between amino or carboxyl groups is the most used. Many natural polymers (such as ε-polylysine (PL), chitosan, hyaluronic acid (HA), sodium alginate (SA), etc.) and DOPA derivatives (3,4-dihydroxyphenethylamine and 3-(3,4-dihydroxyphenyl) propionic acid) contain amino or carboxyl groups. Therefore, the catechol group can be easily incorporated as a pendant group through a coupled reaction. Another reaction capable of the incorporation of the catechol group into a polymer chain is the Schiff base reaction. This method utilizes the Schiff base reactions between the aldehyde polysaccharides and 3,4-dihydroxyphenethylamine [48] or between 3,4-dihydroxybenzaldehyde and amino-containing polysaccharides [49]. Due to the instability of the formed imine bond, the sodium borohydride (NaBH_4_)-mediated reductive amination reaction is usually necessary [49,50]. Using these classic organic reactions, the catechol group has been successfully incorporated into a series of natural polymers, such as PL, HA, chitosan, SA, and gelatin (GT), and synthetic polymers, such as polyethylene glycol (PEG)-based polymers [45,47,51,52,53,54,55].

#### 2.1.2. Polymerization of Catechol-Based Monomers

Catechol is a well-known polymerization inhibitor. Therefore, commonly, catechol is protected before polymerization [47]. Even so, unprotected polymerizations of catechol-based monomers have been reported in recent years [56,57]. The rational regulation of polymerization conditions, such as polymerization time, monomer concentration, and oxygen-free atmosphere, was found to be important for successful polymerization [57]. Unprotected polymerizations of catechol-based monomers have been used in the development of injectable hydrogels. For example, Yang and coworkers used methacrylamide dopamine (DMA) as a catechol-containing monomer and mixed it with polyethylene glycol (PEG) monomethyl ether-modified glycidyl methacrylate-functionalized chitosan and zinc ions to prepare a hydrogel precursor [36]. After initiation by ultraviolet light (365 nm), the monomers were polymerized, and the catechol groups were incorporated into the hydrogels.

#### 2.1.3. Biosynthesis of Catechol-Containing Proteins

Biosynthesis is a method of developing catechol-containing proteins similar to mussel adhesive proteins. In this method, mushroom tyrosinase, which can convert tyrosine residues to DOPA, is used [58]. However, the modification yield is reported to be relatively low. Recently, an in vivo residue-specific incorporation strategy was proposed. This strategy created mussel adhesive proteins with a high DOPA content [59], showing promise in the production of mussel-inspired macromolecules.

### 2.2. Crosslinking Strategy of Mussel-Inspired Injectable Adhesive Hydrogels

Crosslinking among catechol-containing polymers is a necessary step for developing mussel-inspired injectable adhesive hydrogels. Various crosslinking strategies have been developed in recent years. When considering whether the catechol group plays a major role in crosslinking, crosslinking strategies can be divided into catechol-mediated crosslinking and other regular crosslinking.

#### 2.2.1. Catechol-Mediated Crosslinking

**Figure 5:**
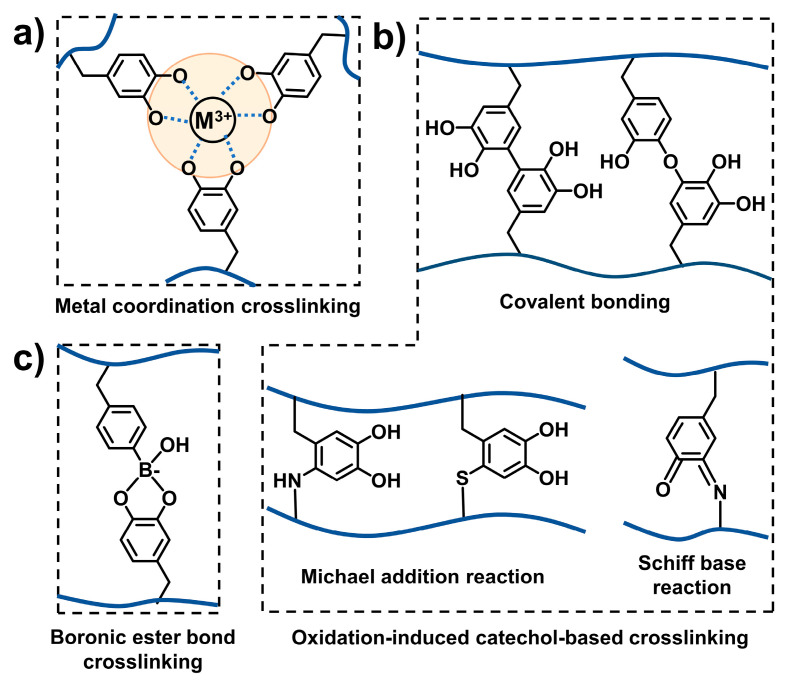
Catechol-mediated crosslinking [2,3,5]. (**a**) Catechol–metal coordination crosslinking. (**b**) Oxidation-induced catechol-based crosslinking. (**c**) Dynamic boron ester-based crosslinking.

The catechol group is usually considered an adhesive group for the improvement of the interfacial adhesion property of materials. In the development of mussel-inspired injectable adhesive hydrogels, the catechol group is also widely used as a functional group for crosslinking of hydrogels (Figure 5). Catechol–metal coordination crosslinking, oxidation-induced catechol-based crosslinking, and dynamic boron ester-based crosslinking are the three main crosslinking ways.

The catechol group in polymers can coordinate with metal ions, such as Fe^3+^, Al^3+^, Zn^2+^, Cu^2+^, and Ti^3+^, to form coordination crosslinking [12]. Among these metal ions, Fe^3+^ is widely studied and used in the preparation of mussel-inspired injectable adhesive hydrogels. Three types of catechol–Fe complexes exist, which are mono-, bis-, and tri-catechol–Fe complexes. The tri-complex is the most stable form [60]. It has been reported that the coordination of catechol and Fe^3+^ is strongly influenced by pH [37,61]. The mono-complex dominates when the pH is lower than 5.6. When the pH is in the range of 5.6–9.1, the bis-complex accounts for a large proportion. Tri-complexes dominate only when the pH is larger than 9.1 [62]. In addition, the molar ratio of catechol to Fe^3+^ influences the coordination types of catechol and Fe^3+^. When the ratio of catechol to Fe^3+^ is high (the amount of Fe^3+^ is low), catechol and Fe^3+^ are prone to forming more tri-complexes [63,64]. Coordination principles have been utilized in the design of mussel-inspired adhesives. For example, Li and coworkers developed a Fe^3+^-crosslinked catechol-modified ε-polylysine adhesive hydrogel. By rationally tuning the pH and Fe^3+^ content in the gelation condition, the resultant adhesive hydrogels exhibited high adhesive strength [65]. In addition, using metal ions with specific functions to crosslink the catechol-containing polymers is found to be effective in developing multifunctional injectable hydrogels. For example, Yang and coworkers utilized the coordination of Zn^2+^ with catechol to develop a composite injectable hydrogel (CSG-PEG/DMA/Zn). Due to the inherent antibacterial property of Zn^2+^, the developed injectable hydrogel exhibited antimicrobial properties [36].

In an alkaline environment, catechol groups are prone to oxidation to form catechol–catechol dimers [13]. This phenomenon has been applied to the development of mussel-inspired hydrogels. Sato and coworkers synthesized a series of catechol-modified hyaluronic acid (HA-CA). They showed that in a weakly alkaline environment (pH 7.4), HA-CA, with a high molar mass and catechol content, was capable of self-assembly into a crosslinked network within about three hours without the addition of additional enzymes or oxidizing agents. This gelation process was mainly due to the oxidation-induced coupling of the catechol groups [66].

Adding oxidizing agents can promote the oxidation of the catechol groups, thus benefiting the gelation process of catechol-containing polymers. For example, Shin and coworkers synthesized catechol-modified hyaluronic acid (HA-CA) and showed that HA-CA can form a hydrogel after the addition of NaIO_4_. Gelation was considered to be induced by the formation of catechol–catechol adducts due to the oxidation of catechol [67]. The incorporation of specific enzymes also contributes to the gelation of catechol-containing polymers by enzymatically crosslinking. For example, Kim and coworkers synthesized catechol-modified poly (γ-glutamic acid) (PGADA). They found that PGADA was able to gel in the presence of horseradish peroxidase (HRP) and hydrogen peroxide (H_2_O_2_) [68]. In addition, in oxidation conditions, the catechol group can transfer to a quinone structure. The formed quinone is capable of reacting with the -NH_2_ group and -SH group, thus improving the crosslinking density [13].

In addition to catechol–metal coordination crosslinking and oxidation-induced catechol-based crosslinking, dynamic boron ester-based crosslinking is used in the preparation of mussel-inspired injectable hydrogels. The boron ester bond is predominantly formed through the complexation of borate with the catechol group, enabling a versatile and robust network formation. For example, Shan and coworkers developed a mussel-inspired polyethylene glycol (PEG)-based hydrogel. The hydrogel contained catechol-modified four-armed PEG (4-arm-PEG-DA) and phenylboronic acid-functionalized four-armed PEG (4-arm-PEG-PBA). At pH 9.0, mixing the two modified PEGs led to the fast formation of a PEG-based hydrogel by boron ester-based crosslinking (borate–catechol complexation) [69].

#### 2.2.2. Other Regular Crosslinking Methods

**Figure 6:**
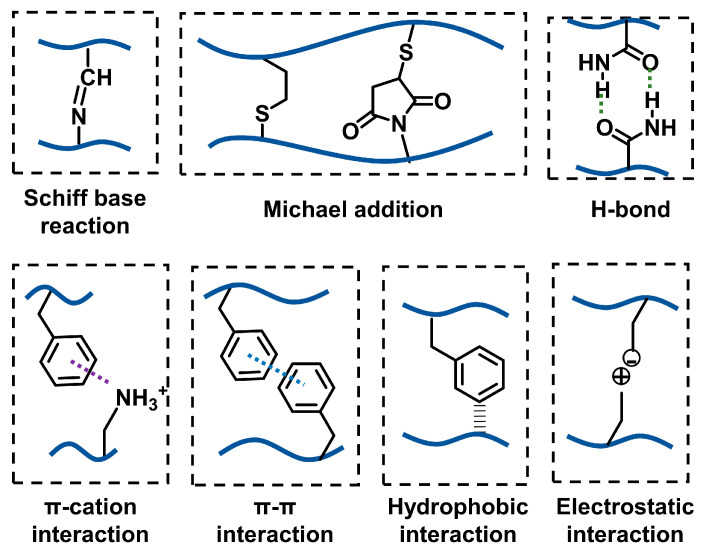
Regular covalent or noncovalent interactions used in the development of injectable hydrogels [3,5,10,12].

Mussel-inspired injectable hydrogels can also be crosslinked by other regular covalent or noncovalent interactions (Figure 6). Covalent crosslinking is the predominant method for developing mussel-inspired injectable gels. The most used reactions in covalent crosslinking include the Schiff base reaction between amino and aldehyde groups and the Michael addition between sulfhydryl and double bonds. For example, Zhou and coworkers synthesized sulfhydryl-modified chitosan (CSS) and catechol and maleimide-functionalized ε-polylysine (Catechol-PL-MAL). After mixing CSS and Catechol-PL-MAL at pH 7.2, the Michael addition between the sulfhydryl in CSS and maleimide in Catechol-PL-MAL induced the gelation of the hydrogel [70]. In this type of gelation, the catechol group was not the major group in crosslinking. Therefore, more catechol groups can participate in interfacial adhesion, which benefits the adhesion of the hydrogel.

Noncovalent interactions are frequently introduced in the design of mussel-inspired adhesive hydrogels. Interactions, including hydrogen bonding, π-π stacking, cation-π interactions, electrostatic forces, and hydrophobic effects, are frequently employed to create self-healable hydrogel networks [3,51]. Compared with covalent bonds, noncovalent interactions are usually weak [71]. Therefore, noncovalent interactions usually work with covalent crosslinking. The introduction of noncovalent interactions into the gel network not only enhances the structural integrity of the hydrogel networks but also endows these materials with additional characteristics, such as self-healing capabilities [72].

#### 2.2.3. Combination of Catechol-Mediated and Other Regular Crosslinking Methods

Mussel-inspired injectable adhesive hydrogels are also able to be prepared by the combination of catechol-mediated and other regular crosslinking methods [61]. Typical examples are summarized in Table 3. The multiple crosslinked structures can endow the hydrogel with enhanced properties. For example, Hu and coworkers developed mussel-inspired hydrogels based on chitosan quaternary ammonium salt (HTCC) and oxidized dextran–dopamine (OD-DA). The hydrogel was double-crosslinked by the catechol–catechol adducts and Schiff base reaction between the aldehyde group in OD-DA and the amino group in HTCC. Owing to the double-crosslinking, the hydrogel exhibited great mechanical properties [73].

## 3. Biomedical Applications of Mussel-Inspired Injectable Adhesive Hydrogels

**Figure 7:**
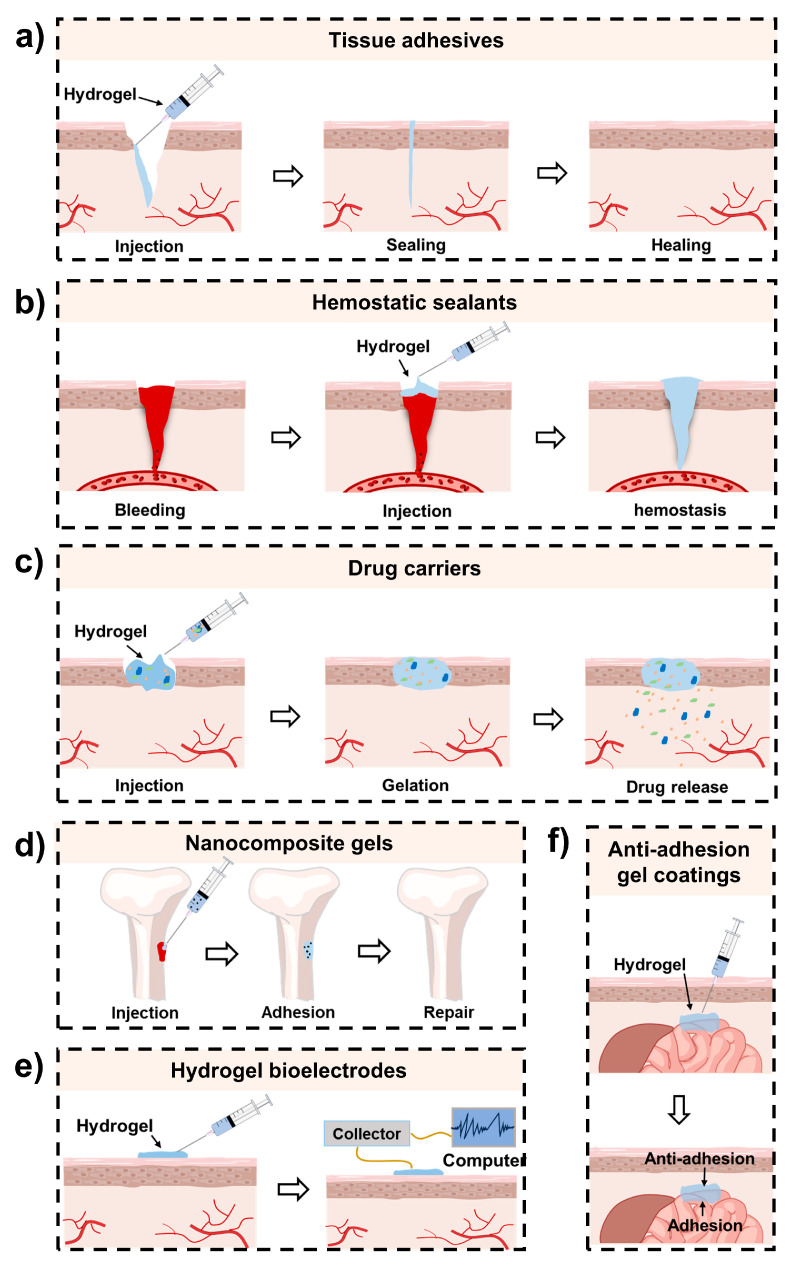
Typical examples of mussel-inspired hydrogels in different areas. (**a**) Tissue adhesives; (**b**) Hemostatic sealants; (**c**) Nanocomposite gels; (**d**) Drug carriers; (**e**) Hydrogel bioelectrodes; (**f**) Anti-adhesion gel coatings.

With the development of adhesive hydrogels, in addition to mussel-inspired adhesive hydrogels, many other types of adhesive hydrogels have been reported for biomedical applications [78,79,80]. Nevertheless, mussel-inspired adhesive hydrogels still show their advantages. First, owing to the existence of the catechol group, mussel-inspired adhesive hydrogels can be easily formed by coordination bonds or other noncovalent interactions. Since these interactions are reversible, the obtained hydrogels usually show self-healing properties [81,82]. Second, the catechol group is a reactive oxygen species (ROS) scavenger. Therefore, the mussel-inspired adhesive hydrogels usually exhibit antioxidant properties [83,84]. Additionally, mussel-inspired polydopamine (PDA)-based nanomaterials possess a photothermal effect; therefore, a hydrogel containing PDA-based nanomaterials usually shows photothermal antibacterial capability [85,86]. With multiple desirable properties, mussel-inspired injectable adhesive hydrogels have been widely applied in numerous biomedical fields. These hydrogels are mainly designed as tissue adhesives [61,87] (for wound closure and healing), hemostatic sealants [75,88] (for hemostasis), nanocomposite gels [41,89] (for bone repair), drug carriers [42,90] (for drug delivery), hydrogel bioelectrodes [44] (for smart sensors), and anti-adhesion gel coatings [45] (for biological coatings) (Figure 7).

### 3.1. Wound Closure and Healing

Millions of wounds, either from accidents or surgery operations, are required to be closed to prevent infection and promote healing each year [91]. In clinical practice, suturing is still the dominant way to close open wounds [13,92]. In recent decades, tissue adhesives have emerged as a new type of tool for wound treatments. The advantages and drawbacks of traditional suturing and tissue adhesive-mediated wound closure are summarized in Table 4. Suturing can stably close wounds. However, it consumes much time and causes secondary damage to tissues surrounding wounds [93,94]. In addition, wounds treated by sutures often have a risk of infection [95] and are prone to leaving scars after healing. Tissue adhesives are capable of closing an open wound by bonding the edges of wound tissues [96]. Compared with clinical sutures, tissue adhesives show advantages in ease of use, sealing of air/fluid leakage, and less pain and scars [97,98]. Therefore, tissue adhesives are becoming attractive alternatives to traditional wound treatment tools. However, current regular tissue adhesives usually show weak mechanical and adhesive strength, and their prices are relatively high.

**Figure 8:**
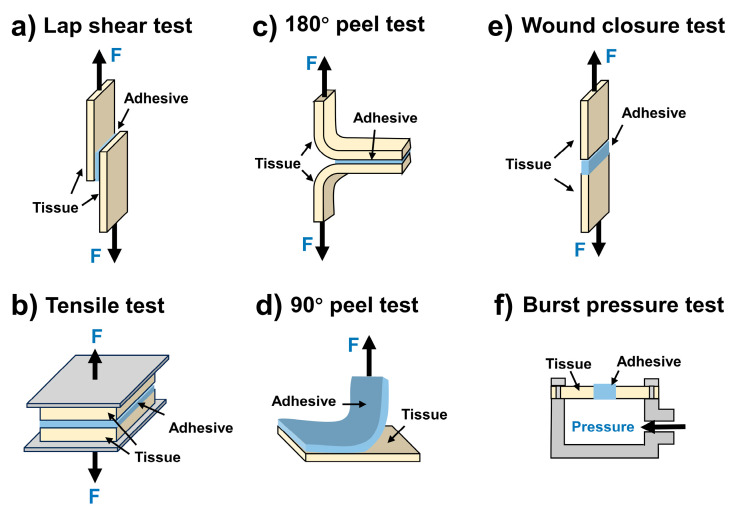
Methods of evaluation of the adhesive performance. (**a**) Lap shear test; (**b**) Tensile test; (**c**) 180° peel test; (**d**) 90° peel test; (**e**) Wound closure test, and (**f**) Burst pressure test.

Due to the ability to adapt to the shapes of wounds, injectable hydrogels have the potential to be used as tissue adhesives. Compared with regular injectable hydrogels, mussel-inspired injectable adhesive hydrogels usually show higher adhesive properties due to the existence of the catechol groups. Their superior adhesive performance has been confirmed by various methods, including the lap shear test, tensile test, 180° peel test, 90° peel test, wound closure test, and burst pressure test (Figure 8). Therefore, mussel-inspired injectable adhesive hydrogels have been widely used as tissue adhesives for wound treatments. For example, Mehdizadeh and coworkers developed an injectable citrate-based mussel-inspired hydrogel bioadhesive (iCMBA) by oxidation-induced crosslinking. The optimized iCMBA bioadhesive showed a strong adhesive strength of about 123 kPa in lap shear tests. Its adhesive strength was about eight times that of commercial fibrin glue. In wound healing experiments, iCMBA hydrogel bioadhesive effectively sealed the wounds on the backs of rats and significantly promoted wound healing [87].

In addition to high adhesive properties, hydrogels for wound treatment should possess high mechanical performance, including toughness and the ability to recover after deformation. The potential of the multiple crosslinking of mussel-inspired hydrogels makes it possible for them to exhibit desirable mechanical performance. Deng and coworkers prepared a mussel-inspired Alg-DA-CATNFC-PAM-Al^3+^DN hydrogel, which was composed of alginate-modified dopamine (Alg-DA), a copolymer of acrylamide and acrylic acid (PAM), and cationized nanofibrillated cellulose (CATNFC). The Alg-DA-CATNFC-PAM-Al^3+^DN hydrogel was crosslinked by triple dynamic interactions, including coordination, hydrogen bonding, and electrostatic interaction, and, therefore, exhibited exceptional mechanical properties and self-healing properties. Upon application at the wound site, the hydrogel effectively closed the 3 cm-long incision on the back of a rat and facilitated wound healing [99]. In addition, the multiple crosslinked structures can endow the hydrogel with multiple functions, thus benefiting wound treatment. Since the catechol group can chelate with Fe^3+^ under weakly alkaline conditions [100], coordination crosslinking is often used in the design of multiple crosslinked hydrogels. For example, Li and coworkers developed a double dynamic bond crosslinked hydrogel adhesive based on catechol-modified ε-polylysine (PL-Cat) and oxidized dextran (ODex). The hydrogel was crosslinked by the dynamic catechol–Fe coordination bond and the dynamic Schiff base bond between the amino group in PL-Cat and the aldehyde group in ODex. This double-dynamic crosslinked characteristic enables the hydrogel to exhibit multiple functions, including on-demand dissolution, repeated adhesion, high adhesive and mechanical properties, injectability, and biocompatibility, which facilitates its application in post-wound closure care [61].

The presence of moisture on tissue surfaces often limits the adhesive strength of traditional hydrogels. It is important for adhesive hydrogels to exhibit robust wet adhesive strength to bond injured tissues. Considering that the marine mussel exhibits robust adhesion to wet rocks, some mussel-inspired adhesive hydrogels were designed and applied in the closure of wet wounds [101]. Recent studies show that the synergistic effect of cation and catechol promoted wet adhesion [102,103]. This principle was applied in developing adhesive hydrogels for wet adhesion. For example, Wang and coworkers developed mussel-inspired catechol-modified ε-polylysine–polyethylene glycol-based hydrogel (PPD hydrogel) by horseradish peroxidase (HRP)-mediated crosslinking. The existence of adjacent catechol–lysine in PPD hydrogel enabled the hydrogel to work in a wet environment. The adhesive strength of optimized PPD hydrogel reached up to about 147 kPa. In vivo studies indicated that the PPD hydrogel was effective in promoting wound repair and skin regeneration [104].

Injured skin is particularly vulnerable to bacterial infections, which result in wound inflammation and delayed healing. The incorporation of DOPA or other catechol compounds endows hydrogels with enhanced adhesion and antimicrobial activity [36,105,106,107]. Therefore, mussel-inspired injectable hydrogel adhesives offer a promising solution for the healing of infected wounds. For example, Guo and coworkers developed antimicrobial mussel-inspired hydrogels based on catechol-functionalized oxidized hyaluronic acid (OHAdop), guar gum (GG), glycol chitosan (GC), borax, and polydopamine nanoparticles (PDA NPs). In addition to their robust tissue adhesive properties, injectability, and self-healing capabilities, due to the photothermal antimicrobial properties of PDA NPs, the developed hydrogels exhibited good antimicrobial properties under near-infrared (NIR) irradiation (808 nm). These desirable properties finally facilitated their application in bacteria-infected wound healing [108].

The healing of diabetic wounds is a significant challenge in clinical practice [73]. Diabetic wounds often involve exacerbated inflammation and an overabundance of reactive oxygen species (ROS) [109,110]. This leads to the leakage of wound tissue fluid, which further fosters pathogen proliferation and results in persistent wound inflammation, non-healing, or recurrence. The development of innovative materials that can effectively relieve the inflammatory response and neutralize ROS is critical for treating diabetic wounds. Since polyphenolic compounds are ROS scavengers, mussel-inspired adhesive hydrogels have the potential to treat diabetic wounds. Fu and coworkers developed a series of tannin–europium coordination complexes (TECs) crosslinked with citrate-based mussel-inspired bioadhesives (TE-CMBAs). The incorporation of TEC improved the antioxidant and anti-inflammatory capabilities of the hydrogel adhesives. In vivo results show that the TE-CMBA hydrogels were able to modulate the inflammatory microenvironment, promote angiogenesis, enhance the production of the extracellular matrix (ECM), and improve re-epithelialization, showing effectiveness in the treatment of diabetic wounds [111].

### 3.2. Hemostasis

Uncontrolled hemorrhage and subsequent wound infection are major problems in trauma treatment [112,113]. Hemorrhage from irregularly shaped and deep wounds is especially severe in emergency care [114]. Nowadays, traditional surgical suturing remains a dominant method for hemostasis. However, it is often criticized for its time-consuming operations and secondary tissue damage [115]. Injectable hydrogel-based tissue adhesives, with their shape adaptability and minimally invasive characteristics, have become promising alternatives for hemorrhage control [116]. Among them, mussel-inspired injectable hydrogels are particularly suitable for hemostasis due to the following reasons: First, hemorrhage control is typically achieved through the formation of a barrier that effectively seals the site of bleeding. A robust hemostatic barrier requires a hemostatic agent with exceptional adhesive strength in the presence of blood and adequate mechanical strength to firmly seal the wound without breakage. Recent studies have shed light on the remarkable effectiveness of catechol for adhesion to wet tissues [117,118]. The multiple crosslinking ways derived from the catechol groups further endow the hydrogels with the potential to be mechanically strong. Therefore, these requirements can be well met by mussel-inspired injectable hydrogels. In addition, the interaction between the catechol group in mussel-inspired hydrogels and reactive residues in proteins or polysaccharides in the blood is known to accelerate the coagulation process [119], which benefits hemostasis. Moreover, the negative charges of polyphenols are capable of activating coagulation factor XII in vivo, initiating a cascade reaction that enhances coagulation [119]. During recent decades, many mussel-inspired injectable hydrogels have been developed for hemorrhage control [120,121]. For example, Fang and coworkers developed a chitosan-based injectable hydrogel, namely, CCS@gel by Schiff base and catechol-mediated crosslinking. Due to the catechol and remnant aldehyde groups, CCS@gel exhibited a remarkable adhesive strength of about 44.9 kPa. In addition, the hydrogel showed high mechanical performance and self-healing properties. In vivo studies show that CCS@gel rapidly covered the injury site and significantly lowered the loss of bleeding [75]. In addition, Hu and coworkers synthesized PVA-DOPA-Cu (PDPC) hydrogels based on poly(vinyl alcohol) (PVA), DOPA, and Cu by coordination crosslinking. The PDPC hydrogels exhibited multiple functions, including high tissue adhesive performance and photothermal and antibacterial properties. After injection at bleeding sites, the PDPC hydrogels can rapidly absorb blood and adhere tightly to the wound, forming an effective hemostatic barrier. They are effective in treating severe hemorrhages from liver injury, carotid artery rupture, and cardiac penetration injury. In addition, owing to the existence of the DOPA/Cu^2+^ complex, PDPC hydrogels also exhibit antimicrobial and anti-inflammatory properties, showing great potential in promoting diabetic wound healing [122].

Nanomaterials, with their large surface area, are capable of generating nanoscale effects that significantly enhance blood absorption and cell adhesion [119]. Moreover, their adjustable physical and chemical properties further facilitate their integration with other hemostatic materials, thereby enhancing hemostatic capabilities. Therefore, mussel-inspired nanocomposite hydrogels have been widely studied and emerged as an essential type in mussel-inspired hemostatic materials [123,124,125]. Xu and coworkers incorporated natural clay nanoparticles (Laponite XLG) into a multifunctional mussel-inspired hydrogel composed of polyacrylic acid (PAA)-based copolymers, oxidized carboxymethylcellulose (OCMC), and tannic acid (TA). The incorporation of Laponite XLG benefited the mechanical properties of the hydrogel. In hemostatic experiments, the hydrogel was found to be capable of reducing hemostasis time and lowering the loss of blood in a liver-bleeding mouse model [126].

Employing materials capable of absorbing blood (or eliminating bodily fluids) and adhering to tissues is a promising therapeutic approach to treating uncontrolled bleeding [127]. Hemostatic powders, with their high surface area and exceptional hygroscopic properties, are often used for hemostasis. However, traditional hemostatic powders have some limitations. For example, they may disperse with the flow of blood and, therefore, have the risk of inducing thrombosis. Additionally, their efficacy in addressing bleeding from deep and incompressible wounds still requires improvement. Shape memory hemostatic sponges offer a novel solution to these issues. These sponges can quickly absorb blood, thereby creating a physical barrier that effectively seals wounds. Dong and coworkers prepared a hydroxyethyl cellulose/soy protein isolate composite sponge (EHSS) and then modified the EHSS with dopamine to obtain a polydopamine-coated injectable hemostatic material (EHP). Silver nanoparticles were homogeneously immobilized on the EHP to finally form the shape memory sponge. Its unique ability to re-expand after absorbing blood allowed the shape memory sponge to serve as a robust physical barrier for stopping bleeding. In vivo results show that the shape memory sponge exhibited remarkable hemostatic performance in a rat liver prick hemorrhage model and other deep, narrow, and noncompressible hemorrhage models [112].

### 3.3. Bone and Cartilage Repair

Bone tissue plays a vital role in bearing weight, shaping physique, and safeguarding vital organs [128]. The occurrence of bone defects due to trauma or tumor removal is common in clinical practice. The reconstruction of such bone injuries is a significant challenge in the medical field. A series of therapeutic strategies have been proposed for bone repair. Autologous and allogeneic bone grafts are the most frequently employed clinical techniques [129,130]. However, there are still some limitations to these treatments, such as the risk of creating new defects at the donor site, potential immune rejection, and the pain associated with surgical procedures [131,132]. In bone tissue engineering, the integration of bioactive growth factors within scaffolds has emerged as an innovative approach to promote bone repair and regeneration [133]. Hydrogels, serving as versatile polymeric scaffolds, are capable of offering robust mechanical support for cell adhesion and can effectively encapsulate bioactive growth factors or cells [133]. Meanwhile, the ideal hydrogel for bone regeneration exhibits exceptional biocompatibility and osteoconductivity. It creates an advantageous microenvironment that fosters cell adhesion, proliferation, and differentiation, which further guides and accelerates the tissue regeneration process [134].

Their robust tissue adhesive properties, minimally invasive abilities, devisable self-healing capabilities, and tunable cell compatibility enable mussel-inspired injectable hydrogels to be applied in bone repair [135,136]. Wang and coworkers developed a mussel-inspired bisphosphorylated injectable composite hydrogel (nHA/PLGA-BP-Dex DC) based on nano-hydroxyapatite (nHA), bisphosphonate–hydrazide-difunctionalized poly(ι-glutamic acid) (PLGA), and aldehyde–catechol-bifunctionalized dextran. The incorporation of catechol and BP groups endowed the composite hydrogel with tissue adhesive properties, self-healing capacity, and osteoconductivity. In vivo cranial bone regeneration experiments confirmed the efficacy of the nHA/PLGA-BP-Dex DC hydrogel in bone repair. Compared with the control group (untreated), the nHA/PLGA-BP-Dex DC hydrogel-treated group showed a higher percentage of bone volume, bone mineral density, and trabecular number [41]. In addition, Ma and coworkers developed mussel-inspired hydrogels (GO-PHA-CPs) based on polydopamine-modified gelatin (Gel-DA), oxidized dextran, polydopamine-functionalized nanohydroxyapatite (PHA), and bioactive cod peptides (CPs). The GO-PHA-CP hydrogels were formed by Schiff base crosslinking and exhibited self-healing abilities, injectability, tissue adhesiveness, antioxidant activity, and osteoinductive properties. In the femoral defect model, GO-PHA-CP hydrogels showed effectiveness in promoting bone regeneration [89].

In addition to nHA, laponite (Lap) [137] was also used with mussel-inspired injectable hydrogels for bone repair. Wu and coworkers developed an injectable and adhesive hydrogel (GMAD/LP) based on gelatin–methacryloyl (GelMA), dopamine-grafted alginate (AD), and polydopamine-functionalized Laponite (Lap@PDA) nanosheets. The GMAD/LP hydrogel exhibited high tissue adhesiveness, robust mechanical strength, injectability, and self-healing abilities. It can serve as an osteoimmune regulator to promote bone regeneration [40].

Cartilage serves as a vital structural tissue, playing a crucial role in minimizing friction, facilitating movement in skeletal joints, and providing structural support across various body regions. Unlike regular bone tissue, however, cartilage lacks blood vessels, cellular reproduction, and regenerative capacity [138]. Chondroitin sulfate (CS), a natural polysaccharide, is usually used as a material to relieve pain and promote cartilage regeneration [139]. Considering the wet adhesion properties of mussels, Zhang and coworkers developed mussel-inspired AD/CS/RSF hydrogels with exceptional adhesion strength on wet surfaces. The AD/CS/RSF hydrogels were prepared by alginate–dopamine (AD), CS, and regenerated silk fibroin (RSF). The hydrogels, after encapsulated exosomes, showed the ability to recruit the migration and inflation of bone marrow stem cells (BMSCs). They also promoted the proliferation and differentiation of BMSCs into chondrocytes, thereby effectively accelerating the healing of cartilage defects in rat knee joints [140].

### 3.4. Drug Delivery

Among many therapeutic strategies, pharmacological intervention is a prevalent and important method. Traditional administration routes, such as oral or intravenous delivery, may sometimes restrict the efficacy of drugs [141]. In recent years, nanodrug delivery systems (NDDSs) have become innovative tools for targeted drug delivery [142]. These nanocarriers possess the ability to deliver therapeutic agents precisely to the intended target. However, conventional nanocarrier systems often have some limitations, including suboptimal targeting accuracy and the risk of premature drug release. One way to improve efficacy is to deliver therapeutic drugs to the target site and release them efficiently. Hydrogels offer a versatile platform for the creation of drug-carrying systems that are responsive to specific stimuli [143]. For decades, hydrogels have been recognized for their critical role in drug delivery to minimize the drawbacks associated with conventional drug delivery systems [144]. Injectable hydrogels are promising in the fields of drug delivery due to their tunable physical and chemical properties, controlled degradation, high water content, and minimally invasive delivery capabilities. Injectable hydrogels usually enable direct drug administration to the target site. In recent years, researchers have developed numerous injectable gels for drug delivery [145,146,147]. Mussel-inspired adhesive hydrogels with high adhesive properties can allow the gels to remain at a specific site for an extended period. Zhao and coworkers developed a catechol-modified chitosan (CMC)- based hydrogel through mussel-inspired coordination crosslinking or oxidation crosslinking. The CMC-based hydrogel exhibited tissue adhesive properties, and its highest adhesive strength was about 9.7 kPa. It was effective as a wound dressing. After being loaded with PNF (a drug from Panax Notoginseng), the CMC-based hydrogel significantly accelerated wound healing [90]. In addition, Gong and coworkers developed dopamine-modified poly(α,β-aspartic acid) injectable hydrogels (PDAEA-Fe^3+^) as a bioadhesive drug delivery system. Owing to the strong binding between dopamine and Fe^3+^, the hydrogels demonstrated excellent adhesive properties. Curcumin was used as a model drug and encapsulated in the hydrogel. The curcumin-loaded PDAEA-Fe^3+^ hydrogel showed a slow and sustained release profile in 4 weeks, suggesting the potential of PDAEA-Fe^3+^ hydrogel in drug delivery [42].

Mussel-inspired composite hydrogels containing nanomaterials are an emerging material that has been widely used in drug delivery [148,149]. Yegappan and coworkers developed a thiol-functionalized hyaluronic acid/polydopamine nanoparticle hydrogel (HA-Cys/PDA hydrogel) by Michael-type addition. This injectable composite hydrogel can release dimethyloxalylglycine (DMOG) drugs sustainably for a period of 7 days. In vitro results indicate that human umbilical vein endothelial cells (HUVECs) treated with DMOG-loaded HA-Cys/PDA hydrogel showed an enhanced capillary tube formation, confirming its potential in tissue engineering [149].

Mussel-inspired injectable gels are also used as versatile delivery platforms for oncology therapeutics. Wu and coworkers developed a unique “Jekyll and Hyde” nanoparticle–hydrogel (NP-gel) based on phenylboronic acid-modified mesoporous silica nanoparticles (PBA-MSNs) and dobutamine-conjugated hyaluronic acid (DOP-HA). The PBA-MSNs can load the anticancer drug doxorubicin (DOX), and DOP-HA can crosslink with PBA-MSNs by acid-cleavable boronate bonds. In the tumor microenvironment, which is mildly acidic and rich in hyaluronidase, DOX-loaded PBA-MSNs are released, and in the adjacent normal tissue, the NP-gel remains dormant. In vivo studies show that the NP-gel can effectively inhibit tumor recurrence and avoid severe toxicity to healthy organs [150]. Additionally, Liu and coworkers developed an injectable hydrogel (Bi_2_Se_3_-DOX@PDA hydrogel) based on polydopamine-coated Bi_2_Se_3_ nanosheets loaded with doxorubicin and dopamine-crosslinked hyaluronic acid (HA-DA) for chemo-photothermal synergistic cancer therapy. The Bi_2_Se_3_-DOX@PDA NSs possess a photothermal effect and can release DOX in a controlled manner. The Bi_2_Se_3_-DOX@PDA hydrogel can be administered precisely via intra-tumoral injection and remains at the site of injection for at least 12 days. It also demonstrates remarkable therapeutic efficacy in T1 xenograft tumors and minimal systemic side effects [151].

### 3.5. Others

Stimuli-responsive hydrogels can respond to a variety of environmental stimuli [152,153] and, therefore, can be applied in the detection of some biological signals. Mussel-inspired hydrogels, with their ability to adhere to biological tissues, are suitable for application in flexible sensors. Pan and coworkers prepared composite hydrogels (PC-CNF-GG-glycerol hydrogels) based on proanthocyanin (PC)-coated cellulose nanofibers (CNFs), guar gum (GG), and glycerol. PCs with phenolic hydroxyl groups improved the adhesion properties of the hydrogels. The borax solution was used as a crosslinking agent, which endows the hydrogel with ion-conducting properties. The strain sensor derived from this hydrogel demonstrated a low-weight detection capability (200 mg) and a fast response time (33 ms). Novel electrodes prepared from this hydrogel can also accurately detect the electrophysiological (EP) signals of humans [44].

Biological coatings can be used to reduce the adhesion between biological tissues [154]. However, conventional preformed hydrogels often fail to fully adhere to irregular wound sites, which have the risk of coating detachment. Mussel-inspired injectable hydrogels with strong adhesive capacity can be designed as gel coatings. For example, Hu and coworkers developed a mussel-inspired hydrogel based on dopamine-functionalized oxidized carboxymethylcellulose (OCMC-DA) and carboxymethyl chitosan (CMCS). The hydrogel was formed in situ on polypropylene (PP) mesh to form modified PP mesh (OCMC-DA/CMCS/PP). Through modification, modified PP meshes exhibited effective anti-adhesion properties in vivo in a rat model, showing their potential to be used as postoperative anti-adhesion materials [45].

## 4. Challenges and Perspectives of Mussel-Inspired Injectable Adhesive Hydrogels

In recent decades, various mussel-inspired injectable adhesive hydrogels have been rationally designed. With their fascinating properties, these hydrogels have gained much attention in many biomedical fields. Although many successes have been achieved, some challenges still exist for mussel-inspired injectable adhesive hydrogels (Table 5). First, the catechol group is prone to oxidation. The oxidation of catechol may reduce the adhesive performance of mussel-inspired hydrogels, especially for some mineral or metal substrates, and is prone to inducing accidental crosslinking [155]. Therefore, commonly, mussel-inspired gels are applied immediately after preparation. The long-term storage of precursor solutions of mussel-inspired hydrogels is usually difficult. There are some strategies to reduce the oxidation of the catechol group. First, utilizing a specific DOPA derivative to fabricate the mussel-inspired hydrogel is effective in lowering the oxidation of the catechol group. For example, methacrylamide dopamine (DMA) can keep catechol stable, even in an oxidation environment [156]. Moreover, the utilization of a reductive group, such as the thiol group, benefits the preservation of the catechol groups. The thiol group has been reported to be able to restore dopa from oxidized dopaquinone [155]. Second, the catechol group is a polymerization inhibitor with the capacity to react with free radicals [47]. On the one hand, this makes the mussel-inspired hydrogel suitable for eliminating ROS in the wound healing process. On the other hand, direct polymerization of catechol-based monomers to form polymers or hydrogels is usually difficult. In general, the catechol group is required to be protected by alkyl silanes and nitrobenzyl before polymerization. However, this method increases the difficulty in the preparation of mussel-inspired materials. Although some studies have successfully polymerized catechol-based monomers by tuning the polymerization conditions [57], the detailed mechanism is still elusive. Efforts can be made to reveal the mechanism of the polymerization of catechol-based monomers. In addition, the catechol group can be used both to enhance the interfacial adhesion and crosslink the hydrogel. Therefore, balancing the content of the catechol groups used for interfacial adhesion and crosslinking the hydrogel is important. Tuning the ratio of catechol to the crosslinking agent or crosslinking hydrogels by other functional groups may be a good solution to this issue [70,100].

## 5. Conclusions

In recent decades, various mussel-inspired injectable adhesive hydrogels have been developed. These adhesive hydrogels combine the advantage of the minimally invasive property of injectable hydrogels and the fascinating properties of mussel-inspired materials. Nowadays, mussel-inspired injectable adhesive hydrogels have been widely applied in many biomedical fields, including wound closure and healing, hemostasis, bone repair, drug delivery, smart sensors, biological coatings, etc. The desirable properties of mussel-inspired injectable adhesive hydrogels facilitate the development of these fields. This review summarizes the design strategies of these mussel-inspired injectable adhesive hydrogels and overviews mussel-inspired injectable hydrogels for biomedical applications. We focus on how the properties of the mussel-inspired injectable hydrogels benefit their applications in these fields. Additionally, the challenges and perspectives of mussel-inspired injectable hydrogels are discussed. We anticipate that this review can provide new inspiration for the design of the next generation of mussel-inspired smart hydrogels.

## Figures and Tables

**Table 1:** Characteristics of preformed hydrogels and in situ injectable hydrogels.

Hydrogel Types	Advantages	Drawbacks
Preformed hydrogels	High physical properties Stable physicochemical properties	Invasive application Fail to fill irregular shapes
Injectable hydrogels	Adaption to irregular shapes Minimally invasive application	Low mechanical properties Weak adhesion properties

**Table 2:** The applications of mussel-inspired glues and some reported examples.

Applications	Examples	Ref.
Wound closure and healing	CSG-PEG/DMA/Zn hydrogel GT-SA-TPF_x_	[36] [37]
Hemostasis	DNAH ^1^ Dopa-OA glue	[38] [39]
Bone repair	GMAD/LP ^2^ nHA/PLGA-Dex hydrogel	[40] [41]
Drug delivery	PDAEA-Fe^3+^ hydrogel QCS/GT/DA	[42] [43]
Smart sensors	PC-CNF-GG-glycerol hydrogel	[44]
Biological coatings	OCMC-DA/CMCS hydrogel	[45]

^1^ DNAH: Double network adhesive hydrogel; ^2^ GMAD/LP: Hydrogel composed of GelMA, AD, and Lap@PDA.

**Table 3:** Combination of catechol-mediated and other regular crosslinking.

Main Crosslinking Methods	Ref.
Schiff base reaction and catechol–Fe coordination	[61]
Schiff base reaction and catechol–catechol adducts	[73]
Schiff base reaction and catechol–Fe coordination/oxidation-induced catechol-based crosslinking	[74]
Schiff base reaction/catechol-based Michael addition and Schiff base reaction	[75]
Fenton reaction and Dopa–Fe^3+^ complexation	[76]
Michael addition and catechol–Fe coordination/oxidation-induced catechol-based crosslinking	[77]

**Table 4:** Characteristics of traditional suturing and tissue adhesives.

Methods	Advantages	Drawbacks
Traditional suturing	Stable wound closure Desirable mechanical features	Time-consuming Secondary tissue damage Risk of wound infection Prone to leaving scars
Tissue adhesives	Easy to manipulation Sealing of air/fluid leakage Minimal tissue damage Less pain and scars	Weak mechanical and adhesive strength Relatively high cost

**Table 5:** The challenges and strategies for addressing challenges.

Challenges	Strategies
Catechol group is prone to oxidation	Utilizing specific DOPA derivative Utilizing reductive group, such as thiol group
Difficulty in polymerization of catechol-based monomers	Protection of catechol groups by alkylsilanes or nitrobenzyl group Tuning the polymerization conditions
Balancing interfacial adhesion and crosslinking	Tuning the ratio of catechol group to crosslinkers Crosslinking by other functional groups

## Data Availability

Not applicable.

## Abbreviations

**Name**	**Abbreviation**
3,4-dihydroxyphenylalanine	DOPA
Dopamine	DA/Dopa
Sodium periodate	NaIO_4_
Hyaluronic acid	HA
Gelatin	Gel/GT
Sodium alginate	SA
ε-polylysine	PL
Polyethylene glycol	PEG
Methacrylamide dopamine	DMA
Poly (γ-glutamic acid)	PGA
Horseradish peroxidase	HRP
Hydrogen peroxide	H_2_O_2_
Sulfhydryl chitosan	CCS
Chitosan quaternary ammonium salt	HTCC
Oxidized dextran	OD
Copolymer of acrylamide and acrylic acid	PAM
Cationized nanofibrillated cellulose	CATNFC
Dextran	Dex
Oxidized dextran	ODex
Oxidized hyaluronic acid	OHA
Guar gum	GG
Gycol chitosan	GC
Reactive oxygen species	ROS
Extracellular matrix	ECM
Poly (vinyl alcohol)	PVA
Polyacrylic acid	PAA
Oxidized carboxymethylcellulose	OCMC
Tannic acid	TA
Nano-hydroxyapatite	nHA
Poly (ι-glutamic acid)	PLGA
Cod peptides	CPs
Laponite	Lap
Chondroitin sulfate	CS
Regenerated silk fibroin	RSF
Dimethyloxalylglycine	DMOG
Human umbilical vein endothelial cells	HUVECs
Doxorubicin	DOX
Proanthocyanin	PC
Cellulose nanofiber	CNF
Carboxymethyl chitosan	CMCS
Polypropylene	PP
Polyethylene glycol monomethyl ether-modified glycidyl methacrylate-functionalized chitosan	CSG-PEG
Protocatechualdehyde	PA
PA-Fe^3+^-Tris	TPF
Oxidized alginate	OA
Gelatin–methacryloyl	GelMA
Dopamine-grafted alginate	AD
Polydopamine-functionalized Laponite	Lap@PDA
Dopamine-modified poly(α,β-aspartic acid) derivative	PDAEA
Quaternized chitosan	QCS
